# Supplementary material for: Adaptive dynamics of memory-one strategies in the repeated donation game
Source: PLoS Comput Biol. 2023 Jun 29;19(6):e1010987. doi: 10.1371/journal.pcbi.1010987 (PMC10337984; doi:10.1371/journal.pcbi.1010987)
Supplement: Attachment — Submitted filename: Rebuttal-Memory-One.pdf [file pcbi.1010987.s001.pdf]

We would like to thank the editor and the three referees for their efforts. The feedback was very helpful and constructive. We have addressed all comments. We believe that the suggested changes have improved the clarity of our manuscript and the impact of our results substantially.
Please find our detailed response below.

Reviewer #1:

This work, taking a fair theoretical approach that seems scientifically healthy, successfully derives that 4D space of memory-1 strategies (denoted by (p_P, p_R, p_S, p_T) where one cooperates with the probability of p_{P,R,S,T} when he obtains {P,R,S,T} in the previous time-step) has an invariant 3D subspace (denoted by (q_0, q_1, q_2) where q_i is the probability to cooperate if i of the two players (focal agent and his opponent) have cooperated in the previous time-step). Qualitatively speaking, 1-memory strategy allows a focal player to evaluate his strategy by the summed-up payoff over past two games, not just by the consequence of a single game. This enables to evolve the so-called ST-reciprocity where one of the two defects while his opponent cooperates (thus, he gains T while the opponent gains S), and subsequently (alternatively in time-direction), he cooperates while his opponent defects (thus, he gains S while the opponent gains T). This is more efficient than R-reciprocity (where both players repeat to cooperate, which brings R to them) if S + T > 2R.
Yet, a donation game (borrowing the authors terminology); Donner & Recipient (D & R) game (which many biologists have favored to call) in other words, where S + T = 2R because of R := b – c, T := b, P := 0, and S := - c) is neutral for whether ST-reciprocity or R-reciprocity being favored.

This MS can be published with the current content, since the authors' methodology supported by several visual results seems solid and reliable. Yet, I would like to suggest the authors to mention about R-reciprocity and ST-reciprocity abovementioned (perhaps with some citations).

***Reply and Changes**: We appreciate the reviewer's observation. Indeed, memory-1 strategies in principle allow for ST-reciprocity (for example, by using a strategy that allows for alternating cooperation like* $\mathbf{p}=(p_1, 0, 1, p_4)$*, with* p_1*,* p_4 *positive but small). We didn't focus on these strategies because the donation game has the property that R-reciprocity is socially optimal (note that in the donation game* $S+T = R < 2R$*). However, we fully agree that games with* $S+T > 2R$ *seem to be understudied in the literature, and now we discuss them in more detail in our revised Model section, together with several references that compare R-reciprocity with ST-reciprocity.*

Reviewer #2:

The iterated Prisoner's Dilemma, along with the corresponding strategies, is a rich and dynamic subject of study and continues to generate interest and new research in various fields, including both evolutionary biology and computer science. The development of sophisticated strategies and the study of their effectiveness in different contexts are also ongoing areas of investigation. Even with the vast amount of research dedicated to the iterated Prisoner's Dilemma and memory-one strategies (which are probably the simplest among all strategies), there are still significant gaps in our understanding of their behavior.

In this paper, the authors discuss the adaptive dynamics of memory-one strategies in the repeated donation game (a particular type of Prisoner's Dilemma satisfying 'equal gains from switching') from an analytical perspective. In particular, they find two interesting mathematical results. First, they show that the four-dimensional space of memory-one strategies contains an invariant three-dimensional subset under adaptive dynamics. The subset is referred to as the collection of 'counting strategies', corresponding to memory-one strategies with p_CD = p_DC. Second, they prove that the adaptive dynamics exhibits a symmetry between orbits froward-in-time and backward-in-time for the donation game. There findings are based on strict mathematical expressions which can be used to characterize the adaptive dynamics among memory-one strategies.

The research presented in this paper represents a noteworthy addition to the literature on Prisoner's Dilemma, as it offers new insights and approaches to understanding the properties of memory-one strategies. And it introduces new avenues for future research on a broader spectrum of games and more general strategy spaces.

However, there are a few issues that need to be addressed to improve its clarity (especially in the mathematics) and quality before it can be accepted for publication.

Reply and Changes*: Thank you very much for this encouraging feedback and your suggestions for improving the clarity of the paper. We have carefully addressed these points, as described below.*

Page 2, lines 43-46: It would be clearer if the authors can add references for each of these strategies, separately. For example, the audience may be interested in knowing in which paper 'random strategy (1/2, 1/2, 1/2, 1/2)' first appeared.

Reply and Changes: *We have added several references for the strategies TFT, GTFT, and WSLS, as well as the random strategy (1/2, 1/2, 1/2, 1/2). Thank you for this useful suggestion.*

Page 6, line 171: How do the authors get the two inequalities of p_CC > c/b and p_DD < 1 – c/b?

Reply and Changes: *Thank you for pointing this out; indeed the paper was missing an explanation for these! We have now added an explicit derivation of these inequalities in the Methods section.*

Page 7, line 224 and Page 13, line 418: There are two different expressions for counting strategies: q = (q_0, q_1, q_2) and q = (q_2, q_1, q_0). The authors need to ensure that the same notations are used consistently throughout.

Reply and Changes: *Thank you for making us aware of the inconsistency! We have now adopted the notation* $\mathbf{q}=(q_2, q_1, q_0)$ *uniformly throughout, to parallel* $\mathbf{p}=(p_{CC}, p_{CD}, p_{DC}, p_{DD})$ *for memory-1 strategies.*

Page 10, Equations: It is somewhat confusing as the correspondences between the variables (x, y, z, w) and the probabilities (p_CC, p_CD, p_DC, p_DD) change. Could it be better that they are fixed? That is, x always corresponds to p_CC, y to p_CD, so on and so forth.

Reply and Changes: *This is a very good point for improving the clarity of the equations. To keep the correspondences fixed, we have now altered the variables in the definitions of the functions f_1, f_2, f_3, and f_4. We have made the same adjustment to the equations for the memory-one counting strategies.*

Page 11, line 269: What is alpha and what is omega? What do these two letters stand for?

Reply and Changes: *In the theory of dynamical systems, the term "ω-limit" is sometimes used to refer to the limit set that a trajectory converges to, forward in time. The term "α-limit" refers to the limit set one gets if one follows the trajectory backward in time. However, the reviewer's comment made us aware that these technical terms are unnecessary. In the revised manuscript, we have replaced them with "limits forward in time" and "limits backward in time," which we hope will improve understanding of this symmetry.*

Other minor points:

The authors may find it more natural to use "memory-one strategies" instead of "memory-1 strategies" throughout the paper.

For consistency, the two letters C and D (representing two actions in the Prisoner's Dilemma) need to be either upright font or italic font from the beginning to the end. The authors may take the time to double check.

Reply and Changes: *Thank you for these good points. We have now adopted "memory-one strategies" and italic font for C and D throughout.*

<u>Reviewer #3:</u>

Memory-one strategies are among the best strategy spaces to study direct reciprocity, but their evolutionary dynamics has been difficult to study analytically. This paper provides an analytical description of the above problem under the framework of adaptive dynamics based on the repeated donation game (prisoner's dilemma). The adaptive dynamics leaves the subspace of counting strategies invariant, and similar invariance occurs in other repeated 2*2 games; The authors of this paper showed a partial characterization of adaptive dynamics for memory-1 strategies and a full characterization for memory-1 counting strategies. The results are meticulous and interesting, and the conclusions are convincing.

I warmly recommend publication after addressing following comments:

Reply: *Thank you for your positive evaluation.*

1. In previous works, the reactive strategies, who only depend on the co-player's previous move, were demonstrated to have some basic properties relative to the memory-one space. It is

recommended that the authors include comparative analysis with previous related works in section Results or Discussion.

Reply and Changes: *We are grateful for this excellent suggestion! In the Methods section, we have now added a new subsection comparing our results with known results concerning the adaptive dynamics of reactive strategies. This is important for contextualizing our results because memory-one strategies exhibit several interesting phenomena not seen in the case of reactive strategies, which we highlight in the new section:*

- *Cooperativity does not always monotonically increase or decrease for memory-one strategies and counting strategies.*
- *The symmetry we describe in Methods behaves more degenerately for reactive strategies, pairing each trajectory with itself. The symmetry becomes much more striking for larger strategy spaces.*

There are also some properties which the two strategy spaces have in common; for example, the set of interior fixed points of adaptive dynamics coincides with the set of equalizer strategies for both reactive and memory-one strategies.
Lastly, we have also added a new figure illustrating the cooperative region for memory-1 strategies, which can be compared to the known cooperative region for reactive strategies.

2. The analytical description of this paper will certainly increase people's understanding of the properties of memory-one strategies, and it is hoped that similar techniques used in this paper can be used to explore more general strategy spaces, such as including conditional cooperators.
3. It is recommended that the authors appropriately summarize important findings on conditional cooperation strategies in section of Introduction.

Reply and Changes: *Thank you for this useful suggestion! We have added a new paragraph to our Introduction which describes conditional cooperation strategies and identifies the most interesting conditional cooperation strategies which can be realized as memory-1 strategies, namely TFT and GTFT. Conditional cooperation strategies have been found to be especially interesting in human behavior, so we point to several references which discuss this in more detail. We indeed hope that techniques similar to those used in this paper can be used to explore more general strategy spaces and more general conditionally cooperative strategies.*
